# Optimal periodic closure for minimizing risk in emerging disease outbreaks

**DOI:** 10.1371/journal.pone.0244706

**Published:** 2021-01-06

**Authors:** Jason Hindes, Simone Bianco, Ira B. Schwartz

**Affiliations:** 1 U.S. Naval Research Laboratory, Washington, DC, United States of America; 2 IBM Almaden Research Center, San Jose, CA, United States of America; INSERM, FRANCE

## Abstract

Without vaccines and treatments, societies must rely on non-pharmaceutical intervention strategies to control the spread of emerging diseases such as COVID-19. Though complete lockdown is epidemiologically effective, because it eliminates infectious contacts, it comes with significant costs. Several recent studies have suggested that a plausible compromise strategy for minimizing epidemic risk is periodic closure, in which populations oscillate between wide-spread social restrictions and relaxation. However, no underlying theory has been proposed to predict and explain optimal closure periods as a function of epidemiological and social parameters. In this work we develop such an analytical theory for SEIR-like model diseases, showing how characteristic closure periods emerge that minimize the total outbreak, and increase predictably with the reproductive number and incubation periods of a disease– as long as both are within predictable limits. Using our approach we demonstrate a sweet-spot effect in which optimal periodic closure is maximally effective for diseases with similar incubation and recovery periods. Our results compare well to numerical simulations, including in COVID-19 models where infectivity and recovery show significant variation.

## 1 Introduction

The COVID19 pandemic, caused by the novel RNA virus SARS-CoV-2 [1], has resulted in devastating health, economic, and social consequences. In the absence of vaccines and treatments, non-pharmaceutical intervention (NPI) strategies have been adopted to varying degrees around the world. Given the nature of the virus transmission, NPI measures have effectively reduced human contacts– both slowing the pandemic, and minimizing the risk of local outbreaks [2, 3]. The use of drastic NPI strategies in China reportedly reduced the basic reproductive number, *R*_0_, to a value smaller than 1, strongly curbing the epidemic within a short period of time [3, 4]. On the other hand widespread testing protocols and contact tracing, in e.g., South Korea, significantly controlled spread during the initial phase of the pandemic [5]. In other countries, the implementation of NPI policies has not been as strict [2], with an optimistic reduction in transmission of roughly a half. To complicate the containment of the disease, early reports indicated significant amounts of pre-symptomatic and asymptomatic transmission [6, 7]. For instance, recent estimates point to asymptomatic infection accounting for around 20–30% of the total, with a similar percentage for pre-symptomatic infections [8]– together producing a majority. These findings have been supported by other experimental studies [9] and analysis of the existing data [10, 11].

As NPI controls such as quarantine, social distancing and testing are enforced, it is important to understand the impact of early release and relaxation of controls on the affected populations [12, 13]. Recent studies have attempted to address how societies can vary social contacts optimally in time in order to maintain economic activity while controlling epidemics [14]. For instance, preliminary numerical studies suggest that periodic closure to control outbreak risk, where a population oscillates between 30-50 days of strict lockdown followed by 30-50 days of relaxed social restrictions, may efficiently contain the spread of COVID-19 and minimize economic damage [15]. These studies test interesting hypotheses, but cannot be immediately generalized to new emerging diseases. A basic understanding of why and when such risk minimizing strategies are effective remains unclear, and may benefit from a general analytical approach.

As a first step in this direction we analyze SEIR-like models with tunable periodic contact rates. Our methods reveal the existence of a characteristic optimal period of contact-breaking between individuals that minimizes the risk of observing a large outbreak, and predicts exactly how such an optimal period depends on epidemic and social parameters. In particular, we show that the optimal period for closure increases (or decreases) predictably with *R*_0_ and the incubation period of a disease, and exists as long as *R*_0_ is below a predictable threshold, and when there is not a time-scale separation between incubation and recovery. We demonstrate analytically that periodic closure is maximally effective for containing disease outbreaks when the typical incubation and recovery periods for a disease are similar—in such cases suppressing large outbreaks with *R*_0_’s as large as 4. Our results compare well to numerical simulations and are robust to the inclusion of heterogeneous infection and recovery rates, which are known to be important for modeling COVID-19 dynamics.

To begin, we first consider the canonical SEIR model with a time-dependent infectious contact rate parameter, *β*(*t*). Individuals in this model are in one of four possible states: susceptible, exposed, infectious, and recovered. Following the simplest mass-action formulation of the disease dynamics, and assuming negligible background births and deaths, the fraction of susceptible (*s*), exposed (*e*), infectious (*i*), and recovered (*r*) individuals in a population satisfy the following differential equations in time (t), where dots denote time derivatives:

$$\dot{s}=-\beta (t)si,$$ (1)

$$\dot{e}=\beta (t)si-\alpha e,$$ (2)

$$\dot{i}=\alpha e-\gamma i,$$ (3)

$$\dot{r}=\gamma i.$$ (4)

Such equations are valid in in the limit of large, well-mixed populations and constitute a baseline description for the spreading of many diseases [16, 17]. Note that *α* is the rate at which exposed individuals become infectious, while *γ* is the rate at which infected individuals recover. If *β*(*t*) = *β*_0_ = constant, it is straightforward to show that the basic reproductive number for the SEIR model, *R*_0_, which measures the average number of new infections generated by a single infectious individual in a fully susceptible population, is *R*_0_ = *β*_0_/*γ* [17–19]. Note in this work when *R*_0_ is written as a constant (no time dependence) it should be taken to mean this value. Typical values for the *R*_0_ of COVID-19 range from 1–4, depending on local population contact rates [4, 20].

## 2 Methods

**Fig 1:**
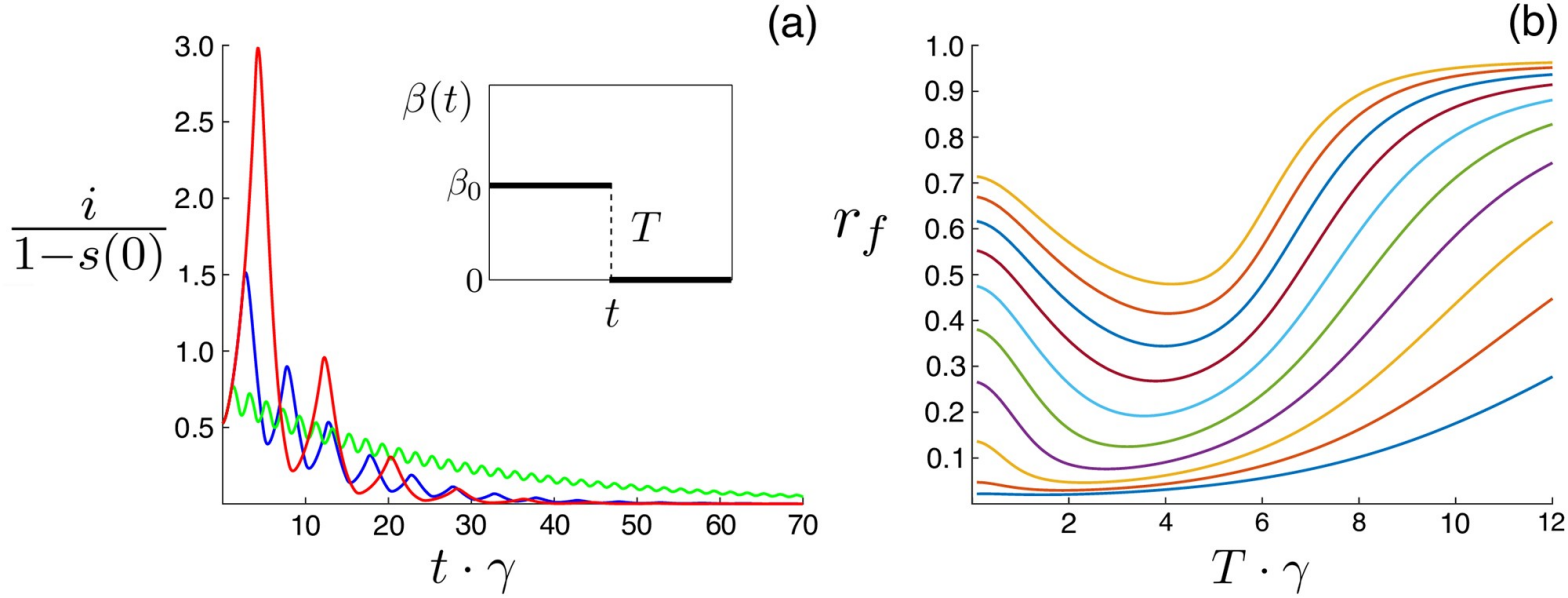
Periodic closure examples. (a) fraction infectious, normalized by initial conditions, versus time for *T* = 10 ⋅ days (green), *T* = 25 ⋅ days (blue), *T* = 40 ⋅ days (red) closure periods. The inlet panel shows a schematic of *β*(*t*). Other model parameters are: *γ*^−1^ = 10 ⋅ days, *α*^−1^ = 8.33 ⋅ days, and $\beta_{0}^{-1}=5\cdot \text{days}$. (b) Outbreak size versus the closure period. Curves correspond to different *R*_0_ = *β*_0_/*γ*, starting from the bottom: first (*R*_0_ = 1.5), second (*R*_0_ = 1.7), …, top (*R*_0_ = 3.3). Other model parameters are identical to (a).

As a simple model for periodic closure we assume a step function for *β*(*t*) with infectious contacts occurring for a period of *T* days with rate *β*_0_, followed by no contacts for the same period, *β*(*t*) = *β*_0_ ⋅ mod(floor{[*t* + *T*]/*T*}, 2) [21]. A schematic of *β*(*t*) is plotted in the inlet panel of Fig 1(a). In S1 Appendix we show results for smoothly varying *β*(*t*) and asymmetric closure, where lockdown and open contacts occur for different amounts of time. It is demonstrated that the results presented in the main text do not qualitatively change under these generalizations. Also in Fig 1(a), we plot an example time-series of the infectious fraction, normalized by the initial fraction of non-susceptibles, for three different closure periods: green (short), blue (intermediate), and red (long). For periods that are not too long or short, the disease remains in a linear spreading regime (as we will show below), and therefore normalizing by the initial conditions gives time series that are initial-condition independent.

Intuitively, since the incubation period, *α*^−1^, is finite, it takes time to build-up infection from small initial values. As a consequence, we expect that it may be possible to allow some disease exposure, before cutting contacts, and the result may be a net reduction in infection at the end of a closure period. For instance, notice that all *i*(*t*) decrease over a full closure cycle, 2*T*, in Fig 1(a). If the closure period is too small, infection can still grow (e.g., as *T* → 0, *R*_0_(*t*) ∼ 〈*R*_0_(*t*)〉_*t*_ = *R*_0_/2 which could be above the epidemic threshold), while if the period is too long, a large outbreak will occur before the control is applied. Between these two limits, there should be an optimal *T* (*T*_min_), that results in a minimum outbreak. To illustrate, in Fig 1(b) we show an example of the final outbreak-size, *r*(*t* → ∞) ≡ *r*_*f*_ starting from *i*(*t* = 0) = 10^−3^, as a function of the closure period for different, equally spaced values of *R*_0_: the bottom curves correspond to smaller values of *R*_0_, while the top curves correspond to larger values.

As expected from the above intuitive argument, simulations show an optimal period that minimizes *r*_*f*_. A natural question is, how does *T*_*min*_ depend on model parameters? Our approach in the following is to develop theory for *T*_min_ in the SEIR-model, and then show how such a theory can be easily adapted to predict *T*_min_ in more complete models, e.g., in COVID-19 models that include heterogeneous infectivity and asymptomatic spread [11, 20].

## 3 Results

### 3.1 Optimal control

It is possible to estimate *T*_min_ by calculating its value in the linearized SEIR model, applicable when the fraction of non-susceptibles is relatively small. When *e*(*t*), *i*(*t*), *r*(*t*), 1 − *s*(*t*) ≪ 1, the dynamics of Eqs (1)–(4) are effectively driven by a 2-dimensional system:

$$\begin{array}{cc} \frac{d\Psi }{dt} & =\gamma M(t)\cdot \Psi , \end{array}$$ (5)

$$\begin{array}{cc} M(t) & =[\begin{array}{cc} -a & R_{0}\left(t\right)\\ \;\;\;a & -1 \end{array}], \end{array}$$ (6)

where *a* ≡ *α*/*γ*, *R*_0_(*t*) ≡ *β*(*t*)/*γ*, and **Ψ**(*t*)^⊤^ = [*e*(*t*), *i*(*t*)].

The first step in calculating *T*_min_ is to construct eigen-solutions of Eqs (5) and (6) in the form

$$\begin{array}{c} \Psi^{p}\left(2T\right)=\nu \left(T\right)\cdot \Psi^{p}\left(0\right), \end{array}$$ (7)

where *ν*(*T*) is the largest such eigenvalue; the superscript *p* denotes the corresponding principal eigenvector. Ignoring the subdominant eigenvalues assumes that after a sufficiently large number of iterations of periodic closure, the dynamics is well aligned with the principle solution no matter what the initial conditions. Unless stated otherwise, simulations are started in this state so that initial-condition effects are minimized. The second step is to calculate the integrated incidence, *r*(2*T*) from the solution of Eq (7), by integrating *i*(*t*) over a full cycle

$$\begin{array}{c} r\left(2T\right)=\int_{0}^{2T}{\left[\Psi^{p}\left(t\right)\right]}_{2}\cdot \gamma dt, \end{array}$$ (8)

where [**Ψ**^*p*^(*t*)]_2_ denotes the infectious-component of **Ψ**^*p*^(*t*). The third step is to calculate the final outbreak size from *r*(2*T*). To this end, it is important to realize that as long as *ν*(*T*) < 1, the outbreak will decrease *geometrically* after successive closure cycles, and therefore *r*_*f*_(*T*) = *r*(2*T*) + *ν*(*T*)*r*(2*T*) + *ν*(*T*)^2^ *r*(2*T*) + …, or

$$\begin{array}{c} r_{f}\left(T\right)=r\left(2T\right)/\left[1-\nu \left(T\right)\right]. \end{array}$$ (9)

Finally, we can find the local minimum of *r*_*f*_(*T*) when *ν*(*T*) < 1 by solving

$$\begin{array}{c} \frac{dr_{f}}{dT}{|}_{T_{\text{min}}}=0. \end{array}$$ (10)

This algorithm gives a single fixed-point equation that determines *T*_min_.

**Fig 2:**
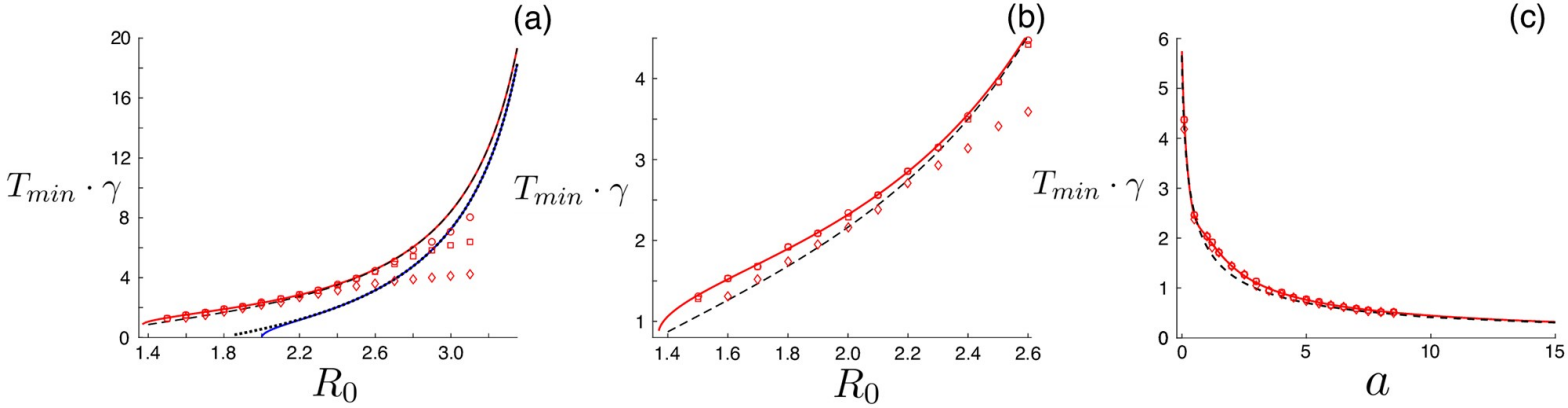
Optimal periodic closure. (a) Period versus *R*_0_ = *β*_0_/*γ*. The solid-red and dashed lines are theoretical predictions (exact and approximate, respectively), and the points are simulation-determined minima for initial fractions infectious: 10^−6^ (circles), 10^−4^ (squares), and 10^−2^ (diamonds). The blue and dotted curves are predictions for the threshold closure period (exact and approximate, respectively). Other model parameters are: *γ*^−1^ = 10 ⋅ days and *α*^−1^ = 8.33 ⋅ days. (b) A refocused version of (a) for smaller values of *R*_0_. (c) Period versus *a* = *α*/*γ*. The color scheme and parameters are identical to (a), except *β*^−1^ = 5.55 ⋅ days.

Since our analysis is based on a piecewise 2-dimensional linear system, it is possible to give every quantity in the previous paragraph an exact expression [22] in terms of epidemiological and social parameters. See S1 Appendix for full derivation and exact expressions for Eqs (7)–(10). Following our procedure gives the prediction curves shown in Fig 2(a). The solid red line indicates the solution to Eq (10), and agrees well with simulation-determined minima of *r*_*f*_(*T*) over a range of *R*_0_ given initial fractions of infectious 10^−6^ (circles), 10^−4^ (squares), and 10^−2^ (diamonds). The simulation-determined minima are computed from *r*_*f*_(*T*) curves like Fig 1(b). It is important to note that our optimal-control theory assumes the validity of the linearized SEIR model, applicable when the total outbreak size, *r*_*f* ≪ 1_. In general, the total outbreak size will increase with the initial fraction of infectious and *R*_0_, and hence, the larger both are, the more simulations will disagree with theory. For example, this explains the better agreement for initial fractions of infectious 10^−6^, as compared to 10^−2^ in Fig 2(b).

On the other hand, the solid blue line in Fig 2(a) indicates the threshold closure period, satisfying

$$\begin{array}{c} \nu (T_{\text{thresh}})=1. \end{array}$$ (11)

The closure period *T*_thresh_ results in the largest eigenvalue of Eqs (5) and (6) equalling unity such that the principal component of exposed and infectious fractions is unchanged after a full closure cycle. If *T* < *T*_thresh_, *ν*(*T*) > 1 and a large outbreak occurs, even with closure, as infection grows over a full cycle for any small non-zero **Ψ**(0). Given this property, *T*_thresh_ gives a lower bound for the optimal period, *T*_min_ > *T*_thresh_. Note: the red curve is always above the blue curve in Fig 2(a).

Before analyzing Eqs (5)–(10) further, we point out two basic dependencies in the (normalized) optimal period *T*_*min*_ ⋅ *γ*. The first is intuitive: as the reproductive number *R*_0_ increases, so does *T*_*min*_ ⋅ *γ*. Hence, the faster a disease spreads the longer a population’s closure-cycle must be in order to contain it. The second is more interesting. Notice in Fig 2(c) that *T*_*min*_ ⋅ *γ* → ∞ as *a* → 0, and *T*_*min*_ ⋅ *γ* → 0 as *a* → ∞. Therefore, recalling *a* = *α*/*γ*, if a disease has a long incubation period, then the optimal closure cycle is similarly long. On the other hand, if a disease has a short incubation period, then the optimal closure cycle is short. In order for periodic closure to be a practical strategy, with *a finite* *T*_*min*_, our results indicate that a∼O(1), roughly speaking, or that the recovery and incubation periods should be on the same time scale– a condition that generally applies to acute infections [19].

Another observation from our approach that we can make is that periodic closure is not an effective strategy for arbitrarily large *R*_0_, as one might expect. One way to see this from the analysis is to notice that the *optimal period diverges for the linear system* at some $R_{0}^{\text{max}}$, as *T*_thresh_ → *T*_min_ → ∞ (at fixed *a*). This transition can be seen in Fig 2(a), as the blue and red curves collide. Above the transition $R_{0}>R_{0}^{\text{max}}$, no periodic closure can keep a disease from growing over a cycle. In this sense $R_{0}^{\text{max}}\left(a\right)$ gives an upper bound on contact rates between individuals that can be suppressed by periodic-closure as a control strategy. We note that an optimal *T*_min_ still exists even when our linear approximation no longer applies, e.g., $R_{0}>R_{0}^{\text{max}}$ (in the sense that *r*(*t* → ∞) is minimized by some *T*_min_), but the benefit of control becomes smaller and smaller as *R*_0_ is increased, and the optimal period becomes increasingly dependent on initial conditions. In such cases, one must resort to numerical simulations of the full non-linear system, Eqs (1)–(4).

A sharper analytical understanding can be found by making the additional approximation that **Ψ**(*t*) ∼ exp[λ_11_ *γt*]**v**_11_, for *t* < *T* and *β*(*t*) = *β*_0_, where

$$\begin{array}{c} \lambda_{11}=\frac{-a-1+\sqrt{{\left(a+1\right)}^{2}+4a\left(R_{0}-1\right)}}{2}. \end{array}$$ (12)

Eq (12) is the largest eigenvalue of **M**(*t*<*T*) with eigenvector **v**_11_. Hence, we ignore the time-decaying part, **Ψ**(*t*)_dec_ ∼ exp[−(*a* + 1 + λ_11_)*γt*]**v**_12_, of a general solution where **v**_12_ is the other eigenvector of **M**(*t*<*T*). Our assumption becomes increasingly accurate with increasing *T*, and Eqs (7)–(11) simplify significantly:

$$\begin{array}{cc} & \nu \left(T\right)\approx e^{T\gamma \lambda_{11}}[\;fe^{-T\gamma }+\left(1-f\right)e^{-Ta\gamma }\;], \end{array}$$ (13)

$$\begin{array}{cc} & \frac{r(2T)}{\bar{r}}\approx \frac{e^{T\gamma \lambda_{11}}-1}{\lambda_{11}}\;\;\;+\\ & \frac{e^{T\gamma \lambda_{11}}}{1-a}(\frac{\left(\lambda_{11}+1\right)\left(1-e^{-T\gamma a}\right)}{a}-\left(a+\lambda_{11}\right)\left(1-e^{-\gamma T}\right)), \end{array}$$ (14)

where

$$\begin{array}{c} f=\frac{{\left(\lambda_{11}+a\right)}^{2}}{\left(a-1\right)\left(2\lambda_{11}+a+1\right)}, \end{array}$$ (15)

and $\bar{r}$ is a constant that depends on *β*_0_, *α*, *γ* and initial conditions, but is independent of *T*. Substituting Eqs (13)–(15) into Eqs (10) and (11) gives a single fixed-point equation for the approximate *T*_min_ and *T*_thresh_ each, which can be easily solved. See S1 Appendix for further details. Examples of the approximate solutions are plotted with dotted and dashed lines in Fig 2, and are almost indistinguishable from the complete linear-theory predictions shown with solid lines.

Using the simplified expressions, we can now show several interesting features of periodic closure. First, since Eqs (13) and (14) are exact for large *T*, we can determine $R_{0}^{\text{max}}$ as a function of *a*. As *T* → ∞, Eq (13) has two scaling limits depending on whether *a* ≥ 1 or *a* < 1. In the former, the second term on the RHS of Eq (13) becomes negligible. As *T* → ∞ the solution of *ν* = 1 is λ_11_ → 1. Solving for *R*_0_ in λ_11_ = 1 gives $R_{0}^{\text{max}}$. Similarly when *a* < 1, as *T* → ∞ the solution of *ν* = 1 is λ_11_ → *a*. Putting the two cases together, gives $R_{0}^{\text{max}}\left(a\right)$, and the phase-diagram for optimal-periodic closure:

$$\begin{array}{c} R_{0}^{\text{max}}=\{\begin{array}{cc} 1+(a+2)/a & \text{if}\;a\geq 1,\\ 2(a+1) & \text{if}\;a<1. \end{array} \end{array}$$ (16)

**Fig 3:**
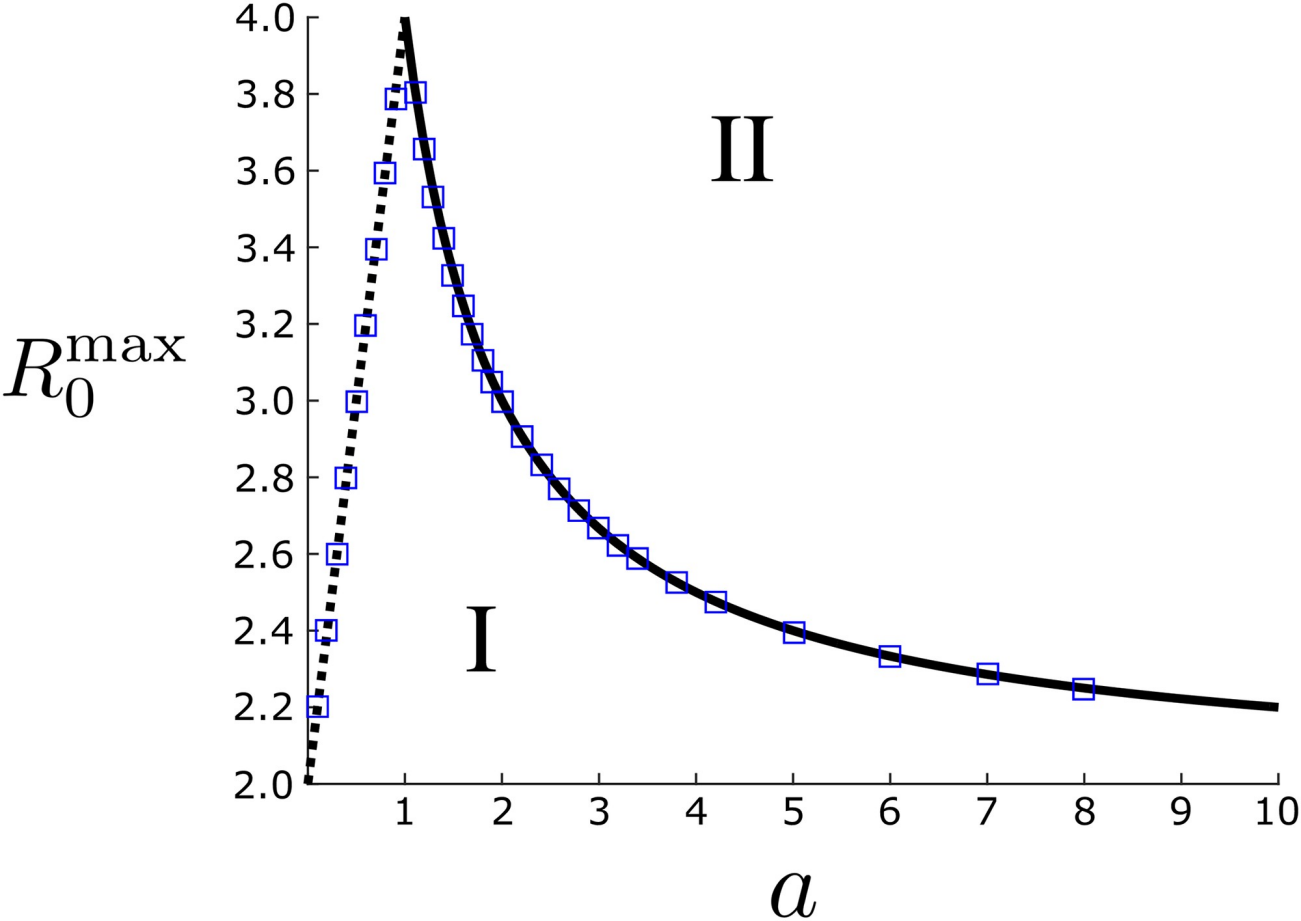
The largest reproductive number *R*_0_ for which periodic closure can keep an SEIR-model disease under threshold. The two regimes are *a* = *α*/*γ* ≥ 1 (solid line) and *a* < 1 (dashed line). Blue squares represent the numerically-determined threshold for the piecewise linear system Eqs (5)–(7) in the long closure-time limit. In region I, outbreaks are contained by optimal closure. In region II, they are not.

Eq (16) is plotted in Fig 3. In region I, the optimal period is predicted to be finite, in which case small outbreaks can be contained by optimal closure. In region II, such outbreaks can not be contained. The blue squares plot numerically-determined thresholds for the piecewise linear system Eqs (5)–(7) in the long closure-time limit. We compute each point by: picking a fixed value of a (starting with *R*_0_ = 2), solving for *T*_thresh_ according to Eqs (5)–(7) and (11), and then repeatedly incrementing *R*_0_ in small steps of 0.001 and solving for *T*_thresh_(*R*_0_; *a*) until it is a large number, i.e., *T*_thresh_(*R*_0_, *a*) *·* = 500. Note that as long as the system Eqs (1)–(4) is below threshold, we can always start with initial fractions of infectious and exposed that are small enough for the linear system to apply.

There are several important cases to notice in Fig 3. The first is that $R_{0}^{\text{max}}$ has a peak when *a* = 1 (*α* = *γ*). The implication is that periodic closure has the largest range of effectiveness, as measured by the ability to keep infection from growing over any closure-cycle, for diseases with *equal* exposure and recovery times. In this symmetric case, periodic closure can prevent large outbreaks as long as *R*_0_ < 4 (compare this to the usual epidemic threshold without closure, *R*_0_ = 1). On the other hand, when there is a time-scale separation between incubation and recovery, *a* → ∞ or *a* → 0, the phase-diagram nicely reproduces the intuitive, time-averaged effective epidemic threshold 〈*R*_0_(*t*)〉_*t*_ = 1, or $R_{0}^{\text{max}}=2$.

### 3.2 COVID-19 model

Now we turn our attention to more complete models that derive from the basic SEIR-model assumptions, but have more disease classes and free parameters which are necessary for accurate predictions. In particular, epidemiological predictions for COVID-19 seem to require an asymptomatic disease state, i.e., a group of people capable of spreading the disease without documented symptoms. Such asymptomatic transmission is thought to be a significant driver for the worldwide distribution of the disease [23, 24], since symptomatic individuals can be easily identified for quarantining while asymptomatics cannot (without widespread testing). Many models have been proposed to incorporate the broad spectrum of COVID-19 symptoms, as well as control strategies such as testing-plus-quarantining [11, 20]. A common feature of such models is the assumption that exposed individuals enter into one of several possible infectious states according to a prescribed probability distribution (e.g., asymptomatic, mild, severe, tested-and-infectious, etc.) with their own characteristic infection rates and recovery times. Following this general prescription, we define *M* infectious classes, *i*_*m*_, where *m* ∈ {1, 2, …*M*}, each with its own infectious contact rate *β*_*m*_(*t*) and recovery *γ*_*m*_ rate, and which appear from the exposed state with probabilities *p*_*m*_. The relevant *heterogeneous* SEIR-model equations become

$$\begin{array}{cc} \frac{de}{dt}=\; & \sum_{m}\beta_{m}\left(t\right)i_{m}s-\alpha e, \end{array}$$ (17)

$$\begin{array}{cc} \frac{di_{m}}{dt}=\; & \alpha p_{m}e-\gamma_{m}i_{m}. \end{array}$$ (18)

Taking a common closure cycle for all individuals in the population, *β*_*m*_(*t*) = *β*_0,*m*_ ⋅ mod(floor{[*t* + *T*]/*T*}, 2) [21], we would like to test our method for predicting *T*_min_ in the more general model Eqs (17) and (18), and demonstrate robustness to heterogeneity. In terms of an algorithm, we could simply repeat our approach for the effective 1 + *M* dimensional linear system; though, we loose analytical tractability. On the other hand, because *T*_min_ is well captured by a linear theory, which depends only on *R*_0_, *a*, and *γ*, we might guess that quantitative accuracy can be maintained for higher dimensional models such as Eqs (17) and (18) by swapping in suitable values for these parameters in our SEIR-model formulas above. This is analogous to the epidemic-threshold condition (*R*_0_ = 1) being maintained in such models, as long as the correct value of *R*_0_ is assumed.

The *R*_0_ for Eqs (17) and (18) is easy to derive using standard methods [17, 18],

$$\begin{array}{c} R_{0}=\sum_{m}p_{m}\beta_{0,m}/\gamma_{m}. \end{array}$$ (19)

Note: the updated *R*_0_ is simply an average over the reproductive numbers for each infectious class. Using this averaging pattern as a starting point, our approach is to substitute the average values of *α*/*γ*_*m*_ and *γ*_*m*_,

$$\begin{array}{c} a=\sum_{m}p_{m}\alpha /\gamma_{m} \end{array}$$ (20)

$$\begin{array}{c} \gamma =\sum_{m}p_{m}\gamma_{m}, \end{array}$$ (21)

into Eqs (7)–(10), or Eqs (13)–(15) for approximate solutions. Namely, for the SEIR model we have an equation 0 = *F*(*R*_0_, *a*, *γ*, *T*_*min*_), where *F* is a function that is determined from Eq (10). Our averaging approximation entails solving the same Eq (10) for *T*_min_, but with parameters given by Eqs (19)–(21).

We point out that this approximation is not arbitrary since in the limit of heterogeneous infectivity only, *γ*_*m*_ = *γ*∀*m*, one solution of Eqs (17) and (18) is *i*_*m*_(*t*) = *p*_*m*_ *i*(*t*), where *i*(*t*) is the total fraction of the population infectious. In this case, the linearized system is still effectively 2-dimensional with parameters *γ*, *α*/*γ*, and *R*_0_, where *R*_0_ is given by Eq (19). For this reason we expect our averaging approximation to be *exact* in the limit of heterogeneous infectivity only, and a good approximation when the variation in recovery rates is not too large.

**Fig 4:**
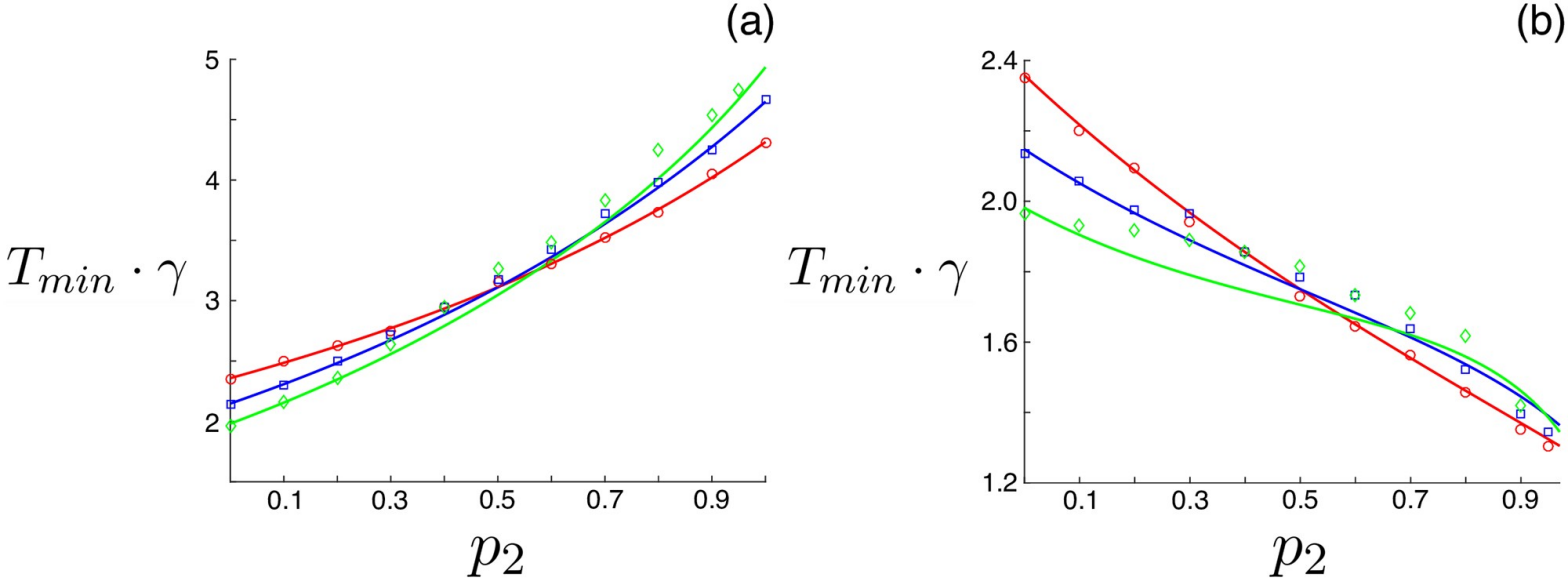
Optimal closure period for a heterogeneous SEIR model with symptomatic and asymptomatic infection as a function of the fraction of asymptomatics. (a) Increased infectivity for asymptomatics, *β*_1_ = 2.1 ⋅ *γ*_1_ and *β*_2_ = 2.6 ⋅ *γ*_2_. The solid lines are theoretical predictions and the points are simulation-determined minima for initial fractions of non-susceptibles 10^−5^. Each series has different recovery times: red ($\gamma_{1}^{-1}=10\cdot \text{days}$, $\gamma_{2}^{-1}=10\cdot \text{days}$), blue ($\gamma_{1}^{-1}=12\cdot \text{days}$, $\gamma_{2}^{-1}=8\cdot \text{days}$), and green ($\gamma_{1}^{-1}=14\cdot \text{days}$, $\gamma_{2}^{-1}=7\cdot \text{days}$). The incubation period is *α*^−1^ = 7 ⋅ days. (b) Decreased infectivity for asymptomatics. Model parameters are identical to (a) except *β*_2_ = 1.5 ⋅ *γ*_2_.

Examples are shown in Fig 4, where each panel shows results for an *M* = 2 model in which asymptomatics are significantly more (a) and less (b) infectious than symptomatics [11]. Symptomatic infectives are denoted with the subscript 1 and asymptomatics with the subscript 2. The optimal closure period is plotted versus the fraction of asymptomatics, *p*_2_. Within each panel the different colors correspond to no variation in recovery rates (red), moderate variation (blue), and large variation (green). Simulation determined *T*_min_ are shown with points and predictions from the averaging theory shown with solid lines. The initial conditions for simulations follow the SEIR model convention– parallel to the principal solution of Eq (7), **Ψ**^*p*^(0)—except that the fraction in each infectious class is *i*_*m*_(0) = *p*_*m*_[**Ψ**^*p*^]_2_. The model parameters were chosen to match similar models [11, 20], which were fit to multiple COVID-19 data sources. As expected, the agreement between theory and simulations ranges from excellent to fair depending on the heterogeneity in recovery rates.

## 4 Discussion

Fig 4 demonstrates that the optimal closure period for COVID-19 can depend significantly on the amount of asymptomatic spread, particularly if there is a large difference in infection rates compared to symptomatic cases. Since asymptomatic spread is difficult to measure directly, especially in the early stages of an emerging disease outbreak, it may be difficult to estimate the optimal control accurately enough for periodic closure to be an actionable strategy on its own. A possible solution is to deploy effective and widespread testing within a population, *early*, and capture the fraction of asymptomatic infections. In any case, if basic parameters are known for an emerging disease dynamics, periodic closure is very effective—producing large reductions in the final outbreak size (e.g., Fig 1(b))– and can be predicted using our methods.

An additional component of population heterogeneity not treated in this work is age dependence, which is known to be particularly important for modelling the COVID-19 pandemic. When considering expanded models that include age compartments, various mixing mechanisms across age groups generate different reproductive rates of infection [25–27]. One extreme compartmented grouping is to decompose a population into young, middle aged, and seniors with age-dependent contact rates between groups, age-dependent recovery periods, and some modest age-dependence in incubation periods. Under weak inter-age mixing assumptions, the result is a system of equations similar to Eqs (17) and (18). As demonstrated in Sec.3.2, the emergence of an optimal periodic control depends primarily on *R*_0_ and the mean incubation period, and persists in spite of population heterogeneity. Although our controls are based on mean epidemiological parameters, it is easy to see how such controls may be distributed across age-dependent groups, and/or spatial clusters. Thus, we expect the inclusion of age-dependent effects to quantitatively change the results presented, but leave our methodology and qualitative findings intact.

Finally, we should remark that in addition to the heterogeneity discussed, parameter fluctuations for COVID-19 spread can occur in space and time. In fact, noise in reporting, differences in local policies, and adherence to the various forms of intervention may cause drastic fluctuations in the local spreading parameters. Given these facts, the well-mixed nature of our model may be insufficient to provide accurate optimal-control predictions. In such cases, a meta-population or network framework may be more appropriate. Yet, the approach that we lay out can be naturally generalized to more accurate and heterogeneous contact-network models, particularly since SIR and SEIR model dynamics on random networks can be described by relatively low-dimensional dynamical systems [28–31], which could be analyzed using the methods described in Sec.2. For small levels of infection, the main contribution from contact heterogeneity is to increase the effective, network *R*_0_. Once the correct *R*_0_ is assumed, however, we expect the network results to be similar to those presented here, though this is a subject for future study.

## 5 Conclusion

In conclusion, a main socio-economic issue with an emerging virus, in the absence of vaccines and treatments, is the enormous damage at all levels of a population. Here we considered a simple approach to model and control an emerging virus outbreak with a finite incubation period. We show that by tuning periodic control of social contact rates, there exists an optimal period that naturally minimizes the outbreak size of the disease, as long as the reproductive number is below a predictable threshold and there is not a time-scale separation between incubation and recovery. Our basic assumption for the existence of such an optimal control rests on early detection of the disease, in which non-susceptible populations are small. Such a basic assumption allows one to analytically predict the optimal period, and provide parameter regions in which an optimal control exists. While in general it has been suggested that periodic closure may help curb the spread of an infectious disease like COVID-19, the implementation of such measures has been, to the best of our knowledge, mostly based on observations of recovery periods and absence of new cases for a given period of time. In this paper, we provide a general formulation that can be utilized to rationally design optimal intervention release protocols. While we start from an SEIR model and expand to heterogeneous models that capture the basic dynamics of COVID-19, our theory can be generally applied to acute infections, with the caveat that recovery and incubation periods should be roughly on the same time scale.

## Supporting information

S1 Appendix Optimal control analysis. Supporting calculations and derivation of the outbreak-minimizing periodic control for the SEIR model. Additional simulation and analysis for both smooth and asymmetric control.
(PDF)
