# Supplementary material for: Optimal periodic closure for minimizing risk in emerging disease outbreaks
Source: PLoS One. 2021 Jan 6;16(1):e0244706. doi: 10.1371/journal.pone.0244706 (PMC7787468; doi:10.1371/journal.pone.0244706)
Supplement: S1 Appendix — Supporting calculations and derivation of the outbreak-minimizing periodic control for the SEIR model. Additional simulation and analysis for both smooth and asymmetric control. (PDF) [file pone.0244706.s001.pdf]

S1 APPENDIX

1. Linear system analysis

In the following, we provide the supporting calculations for the main text, and derive the outbreak-minimizing periodic control for the SEIR model. Note: the citations and equation-references follow the numbering scheme from the main text. To compute eigen-solutions of the linearized SEIR model in the form of Eq.(7), we must first construct the linear transformation

$$\mathbf{\Psi}(2T) = \mathbf{A}\cdot\mathbf{\Psi}(0), \tag{22}$$

where $\mathbf{A}$ is a 2x2 matrix that needs to be determined. A simple way to compute $\mathbf{A}$ is to solve Eqs.(5-6) given unit-vector initial conditions:

$$\mathbf{\Psi}^e(2T) = \mathbf{A}\cdot\begin{bmatrix}1\\0\end{bmatrix}, \tag{23}$$

$$\mathbf{\Psi}^i(2T) = \mathbf{A}\cdot\begin{bmatrix}0\\1\end{bmatrix}, \tag{24}$$

where $\mathbf{\Psi}^e(0)^\top = [1,0]$ and $\mathbf{\Psi}^i(0)^\top = [0,1]$. In this case $\mathbf{A} = [\mathbf{\Psi}^e(2T); \mathbf{\Psi}^i(2T)]$.

The vectors $\mathbf{\Psi}^e(2T)$ and $\mathbf{\Psi}^i(2T)$ can be computed using the two sets of eigen-solutions for the piece-wise linear system Eqs.(5-6). Let us denote the eigenvalues and eigenvectors of $\mathbf{M}(0\le t<T)$, λ_{11}, λ_{12}, $\mathbf{v}_{11}$, and $\mathbf{v}_{12}$:

$$\lambda_{11} = \frac{1}{2}\Big(-a-1+\sqrt{(1+a)^2+4a(R_0-1)}\Big), \tag{25}$$

$$\lambda_{12} = \frac{1}{2}\Big(-a-1-\sqrt{(1+a)^2+4a(R_0-1)}\Big), \tag{26}$$

$$\mathbf{v}_{11} = \begin{bmatrix}(\lambda_{11}+1)/a\\1\end{bmatrix}/\sqrt{1+(\lambda_{11}+1)^2/a^2}, \tag{27}$$

$$\mathbf{v}_{12} = \begin{bmatrix}(\lambda_{12}+1)/a\\1\end{bmatrix}/\sqrt{1+(\lambda_{12}+1)^2/a^2}. \tag{28}$$

Similarly, the eigenvalues and eigenvectors of $\mathbf{M}(T\le t<2T)$ are denoted λ_{21}, λ_{22}, $\mathbf{v}_{21}$, and $\mathbf{v}_{22}$:

$$\lambda_{21} = -1, \tag{29}$$

$$\lambda_{22} = -a, \tag{30}$$

$$\mathbf{v}_{21} = \begin{bmatrix}(\lambda_{21}+1)/a\\1\end{bmatrix}/\sqrt{1+(\lambda_{21}+1)^2/a^2}, \tag{31}$$

$$\mathbf{v}_{22} = \begin{bmatrix}(\lambda_{22}+1)/a\\1\end{bmatrix}/\sqrt{1+(\lambda_{22}+1)^2/a^2}. \tag{32}$$

Starting from any initial-condition vector $\mathbf{x_0}$, the general solution for Eqs.(5-6) when $0\le t<2T$ [22] is

$$\mathbf{x}(0\le t<T) = \sum_{j=1}^{2} a_{1j}(\mathbf{x}_0)e^{\lambda_{1j}t\gamma}\mathbf{v}_{1j}, \tag{33}$$

$$\mathbf{x}(T\le t<2T) = \sum_{j=1}^{2} a_{2j}(\mathbf{x}_0)e^{\lambda_{2j}(t-T)\gamma}\mathbf{v}_{2j}, \tag{34}$$

where

$$a_{11}(\mathbf{x}_0) = \frac{\mathbf{v}_{11}\cdot\mathbf{x}_0-(\mathbf{v}_{11}\cdot\mathbf{v}_{12})(\mathbf{v}_{12}\cdot\mathbf{x}_0)}{1-(\mathbf{v}_{11}\cdot\mathbf{v}_{12})^2}, \tag{35}$$

$$a_{12}(\mathbf{x}_0) = \frac{\mathbf{v}_{12}\cdot\mathbf{x}_0-(\mathbf{v}_{11}\cdot\mathbf{v}_{12})(\mathbf{v}_{11}\cdot\mathbf{x}_0)}{1-(\mathbf{v}_{11}\cdot\mathbf{v}_{12})^2}, \tag{36}$$

$$a_{21}(\mathbf{x}_0) = \frac{a_{11}(\mathbf{x}_0)e^{\lambda_{11}T\gamma}\big(\mathbf{v}_{21}\cdot\mathbf{v}_{11}-(\mathbf{v}_{21}\cdot\mathbf{v}_{22})(\mathbf{v}_{22}\cdot\mathbf{v}_{11})\big)}{1-(\mathbf{v}_{21}\cdot\mathbf{v}_{22})^2} + \frac{a_{12}(\mathbf{x}_0)e^{\lambda_{12}T\gamma}\big(\mathbf{v}_{21}\cdot\mathbf{v}_{12}-(\mathbf{v}_{21}\cdot\mathbf{v}_{22})(\mathbf{v}_{22}\cdot\mathbf{v}_{12})\big)}{1-(\mathbf{v}_{21}\cdot\mathbf{v}_{22})^2}, \tag{37}$$

$$a_{22}(\mathbf{x}_0) = \frac{a_{11}(\mathbf{x}_0)e^{\lambda_{11}T\gamma}\big(\mathbf{v}_{22}\cdot\mathbf{v}_{11}-(\mathbf{v}_{21}\cdot\mathbf{v}_{22})(\mathbf{v}_{21}\cdot\mathbf{v}_{11})\big)}{1-(\mathbf{v}_{21}\cdot\mathbf{v}_{22})^2} + \frac{a_{12}(\mathbf{x}_0)e^{\lambda_{12}T\gamma}\big(\mathbf{v}_{22}\cdot\mathbf{v}_{12}-(\mathbf{v}_{21}\cdot\mathbf{v}_{22})(\mathbf{v}_{21}\cdot\mathbf{v}_{12})\big)}{1-(\mathbf{v}_{21}\cdot\mathbf{v}_{22})^2}. \tag{38}$$

In particular, we have the following expressions for $\mathbf{\Psi}^e(2T)$ and $\mathbf{\Psi}^i(2T)$:

$$\mathbf{\Psi}^e(2T) = \sum_{j=1}^{2} a_{2j}(\mathbf{\Psi}^e(0))e^{\lambda_{2j}T\gamma}\mathbf{v}_{2j}, \tag{39}$$

$$\mathbf{\Psi}^i(2T) = \sum_{j=1}^{2} a_{2j}(\mathbf{\Psi}^i(0))e^{\lambda_{2j}T\gamma}\mathbf{v}_{2j}. \tag{40}$$

Next, the principal eigenvalue of the 2x2 matrix $\mathbf{A}$ is

$$\nu(T) = \frac{1}{2}\Big([\mathbf{\Psi}^e(2T)]_1+[\mathbf{\Psi}^i(2T)]_2+\sqrt{([\mathbf{\Psi}^e(2T)]_1+[\mathbf{\Psi}^i(2T)]_2)^2-4([\mathbf{\Psi}^e(2T)]_1[\mathbf{\Psi}^i(2T)]_2-[\mathbf{\Psi}^e(2T)]_2[\mathbf{\Psi}^i(2T)]_1)}\Big), \tag{41}$$

with a corresponding principal eigenvector,

$$\mathbf{\Psi}^p(0) = \begin{bmatrix} 1 \\ \frac{\nu(T)-[\mathbf{\Psi}^e(2T)]_1}{[\mathbf{\Psi}^i(2T)]_1}. \end{bmatrix}. \quad (42)$$

Equations (41-42) derive from standard formulae for 2x2 matrices[22]. Note: $\mathbf{\Psi}^p(0)$ in Eq.(42) is unnormalized.

Now that we have $\mathbf{\Psi}^p(0)$, we simply integrate the infective-component of the principal vector

$$\mathbf{\Psi}^p(0 \le t < T) = \sum_{j=1}^{2} a_{1j}(\mathbf{\Psi}^p(0)) e^{\lambda_{1j} t\gamma} \mathbf{v}_{1j}, \quad (43)$$

$$\mathbf{\Psi}^p(T \le t < 2T) = \sum_{j=1}^{2} a_{2j}(\mathbf{\Psi}^p(0)) e^{\lambda_{2j}(t-T)\gamma} \mathbf{v}_{2j}, \quad (44)$$

over a full closure cycle per Eq.(8). The total outbreak-size in the long-time limit then follows easily from Eqs.(8-9), and is given by

$$r_f(T) = \frac{\sum_{i=1}^2 \sum_{j=1}^2 a_{ij}(\mathbf{\Psi}^p(0))[\mathbf{v}_{ij}]_2 (e^{\lambda_{ij} T\gamma} - 1)/\lambda_{ij}}{1-\nu(T)}. \quad (45)$$

Taking the derivative of Eq.(47) with respect to T and setting it equal to zero, as directed by Eq.(10), gives a transcendental equation for T_{min} that can be solved using standard numerical methods.

Finally, to derive the approximate solutions, Eqs.(13-15), the above is repeated assuming $a_{12}(\Psi^p(0)) = 0$ (or $e^{\lambda_{12} t\gamma} \to 0$). As stated in the main text, this is equivalent to assuming only exponential growth of exposed and infectious fractions for $0 \le t < T$.

2. *Smooth periodic closure*

In this section we show numerical evidence that our analysis is robust to smoothing the square-wave control of $\beta(t)$, especially for smaller values of R_0 and moderate levels of smoothing. In particular, we consider the following periodic function

$$\beta(t) = \frac{\beta_0}{2}\left(\frac{\tan^{-1}\{\sin(2\pi t/2T)/\delta\}}{\tan^{-1}\{1/\delta\}} + 1\right), \quad (46)$$

where the smoothness parameter, δ, interpolates between the square-wave for $\delta = 0$ (where our analysis is performed), and a sine-wave for $\delta = 1$. Figure 5 (a) shows examples of $\beta(t)$ for small (red-dashed), moderate (blue-dotted), and large (green-solid) values of δ. We can see that effectively the new time scale, $T\delta$, introduces an inertia to the controls whereby the the min and max of $\beta(t)$ take an additional time $\mathcal{O}(T\delta/2)$ to appear, compared to the $\delta = 0$ limit.

The corresponding simulation values of $T_{\rm min}$ are plotted in Fig.5 (b), assuming Eq.(46). We can see that for a significant range of δ values, theory and simulations agree as long as R_0 is not too large. For instance, disagreement becomes noticeable near $R_0 \approx 2.5$ for $\delta = 0.25$ and $R_0 \approx 2.7$ for $\delta = 0.1$, both with an initial infectious fraction of $\mathcal{O}(10^{-4})$. From these results we can conclude that the faster complete lock-down can be implemented ($\delta \to 0$), the better agreement becomes, and the more effective it is.

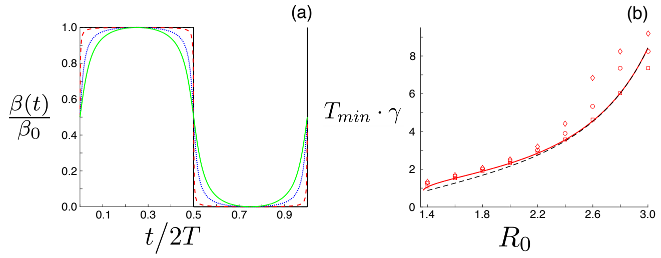

FIG. 5. Smooth periodic control. (a) Three examples of smooth $\beta(t)$, Eq.(46), when $\delta = 0.01$ (red-dashed), $\delta = 0.1$ (blue-dotted), and $\delta = 0.25$ (green-solid). (b) Corresponding values of $T_{\rm min}$ as a function of R_0. The solid-red and dashed lines are theoretical predictions for the square-wave control (exact and approximate, respectively). Points are simulation-determined minima for initial fractions infectious 10^{-4}, and $\delta = 0.01$ (squares), $\delta = 0.1$ (circles), and $\delta = 0.25$ (diamonds). Other model parameters are: $\gamma^{-1} = 10\cdot$days and $\alpha^{-1} = 8.33\cdot$days.

Since the smooth variation in contact rates is more realistic in terms of actual implementation in society, these results suggest that the control theory developed is mainly applicable for R_0's that are not too large. However, we note that since our analysis relied on the validity of the linearized SEIR model (applicable for relatively small exposed and infectious fractions), we already expected simulation-theory disagreement to appear for larger values of R_0, as stated in the main text – at least for realistic levels of initial infection. Hence, the practical domain of application for theory is not significantly changed by the new smoothness results.

3. *Asymmetric closure*

Lastly, in this section we demonstrate that it is straightforward to generalize our methods to asymmetric closure. For instance, let us assume that infection occurs over a reduced period $T_1 \equiv \epsilon T$, where $\epsilon \le 1$, and no infection over a closure period $T_2 \equiv (2-\epsilon)T$. All of the linear-analysis in App. 1 is the same, except $T \to T_1$, in Eqs.(37-38), and $T \to T_2$ in Eqs.(39-40). Substituting these updated formulas into Eqs.(41-42) we get the updated, final recovery-fraction,

$$r_f(T) = \frac{\sum_{i=1}^2 \sum_{j=1}^2 a_{ij}(\mathbf{\Psi}^p(0))[\mathbf{v}_{ij}]_2 (e^{\lambda_{ij} T_i\gamma} - 1)/\lambda_{ij}}{1-\nu(T)}. \quad (47)$$

Taking the derivative of Eq.(47) with respect to T and setting it equal to zero, gives a transcendental equation for T_{min}, just as in the symmetric case.

Figure 6 shows an example of such asymmetric control when $\epsilon = 0.8$. In the left panel $\beta(t)$ is plotted. In the right panel, two examples are compared for $T_{\min}(R_0)$. The dashed-red line plots numerical predictions for the local minimum of Eq.(47) when $\epsilon = 0.8$; the solid-red line plots the same for $\epsilon = 1.0$, i.e, the symmetric control. Circle and square points indicate simulation-determined optimal-control periods. As in the symmetric case, we can see that the linear-theory predicts the optimal control accurately as long as R_0 is not too large (i.e., the final outbreak is sufficiently small). In addition, the asymmetric closure is able to control epidemics for a significantly larger range of R_0's, compared to the symmetric case; this is a consequence of infection occurring at a reduced period. These results support the idea that asymmetric closure is a better control strategy for minimizing outbreaks of emergent diseases with high infectivity.

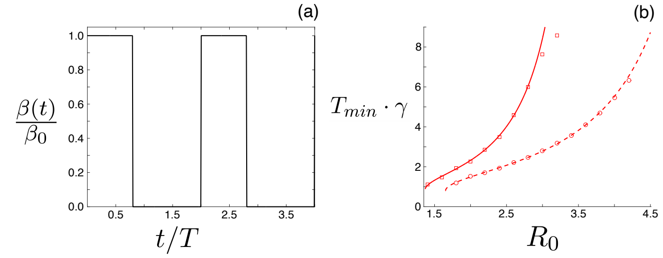

FIG. 6. Asymmetric control. (a) Example of $\beta(t)$ in which infection occurs over a reduced period, ϵT, and closure over a period $(2-\epsilon)T$, where T is a free parameter. (b) Optimal control period as a function of R_0. The solid-red and dashed lines are theoretical predictions for $\epsilon = 1.0$ and $\epsilon = 0.8$, respectively. Points are simulation-determined minima for $\epsilon = 1.0$ (squares) and $\epsilon = 0.8$ (circles), for initial fractions infectious 10^{-5}. Other model parameters are: $\gamma^{-1} = 10 \cdot$days and $\alpha^{-1} = 8.33 \cdot$days.
